# Olaparib in recurrent isocitrate dehydrogenase mutant high-grade glioma: A phase 2 multicenter study of the POLA Network

**DOI:** 10.1093/noajnl/vdae078

**Published:** 2024-05-27

**Authors:** Ines Esparragosa Vazquez, Marc Sanson, Olivier L Chinot, Maxime Fontanilles, Romain Rivoirard, Laure Thomas-Maisonneuve, Stéphanie Cartalat, Emeline Tabouret, Romain Appay, Alice Bonneville-Levard, Amélie Darlix, David Meyronet, Marc Barritault, François Gueyffier, Laurent Remontet, Delphine Maucort-Boulch, Jérôme Honnorat, Caroline Dehais, François Ducray, POLA Network, C Desenclos, N Guillain, P Menei, A Rousseau, T Cruel, S Lopez, M Abad, N Hamdan, C Adam, F Parker, R Seizeur, I Quintin-Roué, G Chotard, C Bronnimann, D Ricard, C Godfraind, T Khallil, D Cazals-Hatem, T Faillot, C Gaultier, M C Tortel, I Carpiuc, P Richard, H Aubriot-Lorton, F Ghiringhelli, A Djelad, C A Maurage, E M Gueye, F Labrousse, F Ducray, D Meyronet, D Figarella-Branger, O Chinot, L Bauchet, V Rigau, G Gauchotte, L Taillandier, M Campone, D Loussouarn, V Bourg, F Vandenbos-Burel, J-S Guillamo, P Roger, C Blechet, H Adle-Biassette, F Bielle, A Carpentier, C Dehais, S Milin, M Wager, P Colin, M D Diebold, D Chiforeanu, E Vauleon, F Marguet, O Langlois, F Forest, M J Motso-Fotso, M Andraud, B Lhermitte, G Noel, M Bernier, N Younan, C Rousselot-Denis, I Zemmoura, C Joubert, E Cohen-Moyal, E Uro-Coste, F Dhermain

**Affiliations:** Department of Neuro-Oncology, East Group Hospital, Hospices Civils de Lyon, Lyon, France; Sorbonne Université, Inserm, CNRS, UMR S 1127, Paris Brain Institute (ICM), Paris, France; Service de Neurologie 2, AP-HP, Hôpital de la Pitié-Salpêtrière, Paris, France; Aix-Marseille University, CNRS, Inst Neurophysiopathol, Marseille, France; Department of Neuro-Oncology, AP-HM, University Hospital Timone, Marseille, France; Department of Medical Oncology, Cancer Centre Henri Becquerel, Rouen, France; UNIROUEN, Inserm U1245, IRON group, Normandy Centre for Genomic and Personalized Medicine, Normandie university, Rouen University Hospital, Rouen, France; Oncology Department, CHU de Saint-Etienne, Saint Etienne, France; Department of Neuro-Oncology, East Group Hospital, Hospices Civils de Lyon, Lyon, France; Department of Neuro-Oncology, East Group Hospital, Hospices Civils de Lyon, Lyon, France; Aix-Marseille University, CNRS, Inst Neurophysiopathol, Marseille, France; Department of Neuro-Oncology, AP-HM, University Hospital Timone, Marseille, France; Aix-Marseille University, CNRS, Inst Neurophysiopathol, Marseille, France; Department of Pathology, AP-HM, University Hospital Timone, Marseille, France; Department of Medical Oncology, Leon Bérard Cancer Centre, Lyon, France; Department of Medical Oncology, Institut Régional du Cancer de Montpellier, Institut de Génomique Fonctionnelle, CNRS, INSERM, University of Montpellier, Montpellier, France; LabEx Dev2CAN, Institut Convergence Plascan, Centre de Recherche en Cancérologie de Lyon, Inserm U1052, CNRS UMR5286, Université de Lyon, Université Claude Bernard Lyon 1, Centre Léon Bérard, CEDEX 08, Lyon, France; Department of Pathology, East Group Hospital, Hospices Civils de Lyon, Lyon, France; LabEx Dev2CAN, Institut Convergence Plascan, Centre de Recherche en Cancérologie de Lyon, Inserm U1052, CNRS UMR5286, Université de Lyon, Université Claude Bernard Lyon 1, Centre Léon Bérard, CEDEX 08, Lyon, France; Department of Pathology, East Group Hospital, Hospices Civils de Lyon, Lyon, France; Pôle de Santé Publique, Hospices Civils De Lyon, Lyon, France; Biostatistics-Bioinformatics Department, Public Health Unit. Hospices Civils de Lyon, Lyon, France; Biostatistics-Bioinformatics Department, Public Health Unit. Hospices Civils de Lyon, Lyon, France; Department of Neuro-Oncology, East Group Hospital, Hospices Civils de Lyon, Lyon, France; MeLiS - UCBL-CNRS UMR 5284-INSERM U1314, Université Claude Bernard Lyon 1, Lyon, France; Service de Neurologie 2, AP-HP, Hôpital de la Pitié-Salpêtrière, Paris, France; Department of Neuro-Oncology, East Group Hospital, Hospices Civils de Lyon, Lyon, France; LabEx Dev2CAN, Institut Convergence Plascan, Centre de Recherche en Cancérologie de Lyon, Inserm U1052, CNRS UMR5286, Université de Lyon, Université Claude Bernard Lyon 1, Centre Léon Bérard, CEDEX 08, Lyon, France

**Keywords:** clinical trial, high-grade gliomas, IDH, olaparib

## Abstract

**Background:**

Based on preclinical studies showing that IDH-mutant (IDHm) gliomas could be vulnerable to PARP inhibition we launched a multicenter phase 2 study to test the efficacy of olaparib monotherapy in this population.

**Methods:**

Adults with recurrent IDHm high-grade gliomas (HGGs) after radiotherapy and at least one line of alkylating chemotherapy were enrolled. The primary endpoint was a 6-month progression-free survival rate (PFS-6) according to response assessment in neuro-oncology criteria. Pre-defined threshold for study success was a PFS-6 of at least 50%.

**Results:**

Thirty-five patients with recurrent IDHm HGGs were enrolled, 77% at ≥ 2nd recurrence. Median time since diagnosis and radiotherapy were 7.5 years and 33 months, respectively. PFS-6 was 31.4% (95% CI [16.9; 49.3%]). Two patients (6%) had an objective response and 14 patients (40%) had a stable disease as their best response. Median PFS and median overall survival were 2.05 and 15.9 months, respectively. Oligodendrogliomas (1p/19q codeleted) had a higher PFS-6 (53.4% vs. 15.7%, *P* = .05) than astrocytomas while an initial diagnosis of grade 4 astrocytoma tended to be associated with a lower PFS-6 compared to grade 2/3 gliomas (0% vs 31.4%, *P* = .16). A grade 2 or 3 treatment-related adverse event was observed in 15 patients (43%) and 5 patients (14%), respectively. No patient definitively discontinued treatment due to side effects.

**Conclusions:**

Although it did not meet its primary endpoint, the present study shows that in this heavily pretreated population, olaparib monotherapy was well tolerated and resulted in some activity, supporting further PARP inhibitors evaluation in IDHm HGGs, especially in oligodendrogliomas.

**Key Points**

- Olaparib was well tolerated in patients with recurrent IDH-mutant high-grade gliomas.
- The OLAGLI trial supports further evaluation of PARP inhibitors in IDH-mutant high-grade gliomas.
- Oligodendrogliomas could be more sensitive to PARP inhibition than astrocytomas.

**Importance of the Study**

IDH-mutant high-grade gliomas that recur after radiotherapy and chemotherapy represent an unmet medical need. There is strong preclinical evidence supporting the therapeutic potential of PARP inhibitors in IDH-mutant tumors. The present phase 2 clinical trial shows that olaparib monotherapy is associated with some activity in heavily pretreated recurrent IDH-mutant high-grade gliomas, and supports further evaluation of PARP inhibitors in this population. Oligodendrogliomas seemed to benefit the most from olaparib while no benefit was observed in grade 4 astrocytomas.

Adult isocitrate dehydrogenase (IDH, 1 or 2) mutant high-grade gliomas (IDHm HGGs) consist of grade 3 IDHm and 1p/19q codeleted oligodendrogliomas, and grade 3/4 IDHm astrocytomas.^1,2^ Although their prognosis is much better than that of IDH wild-type glioblastomas, most IDHm HGGs recur despite an initial treatment consisting of maximal safe surgical resection followed by radiotherapy and adjuvant alkylating chemotherapy (temozolomide [TMZ] or PCV [procarbazine, CCNU, vincristine]). At the time of recurrence, most patients receive alkylating chemotherapies (nitrosourea- or TMZ-containing regimens); however, these treatments have modest efficacy.^2–6^ IDH inhibitors have demonstrated efficacy in low-grade IDHm gliomas but do not seem to be effective (at least as monotherapy) in recurrent IDHm HGGs which are most frequently contrast-enhancing tumors, a characteristic that has been negatively associated with the response to these treatments.^7,8^ In 2017, Sulkowski et al. provided preclinical evidence that 2-hydroxyglutarate, the oncometabolite resulting from the mutant IDH protein, suppresses the homology-dependent DNA repair pathway, conferring to IDHm tumors a “BRCAness” phenotype and a sensitivity to poly (adenosine5ʹ-diphosphate-ribose) polymerase (PARP) inhibitors, as in BRCA mutant tumors.^9^ Based on these results several studies were launched to evaluate the efficacy of PARP inhibitors in IDHm gliomas.^10^ Herein, we present the results of an open-label multicenter phase 2 study to evaluate the efficacy of olaparib in a population of patients with recurrent IDHm HGGs after radiotherapy and at least one line of alkylating chemotherapy.

## Materials and Methods

### Study design

The Olaparib in Recurrent IDH-mutant Glioma (OLAGLI) trial was an open-label multicenter single-arm phase 2 study. Patients were recruited at 6 POLA (Prise en charge des Oligodendrogliomes Anaplasiques) Network centers. The POLA Network is a French network dedicated to care and research in IDHm HGGs.^11^ The study was planned to last 30 months (inclusion period: 18 months, follow-up after the inclusion of the last patient: 12 months). The trial was registered in the European Clinical Trials Database under the accession number EUDRACT 2018-002584-25 and in ClinicalTrials.gov under the accession number NCT03561870. It was approved by the French National Medicines agency and an ethic committee (*Comité de protection des personnes Sud-Ouest et Outre-Mer 1)* according to French legislation. The study was conducted in accordance with the Declaration of Helsinki and Good Clinical Practice. All patients gave written informed consent prior to enrollment.

### Sample size

Based on phase 2 trials demonstrating a PFS-6 of 29% to 51% in recurrent IDHm HGGs treated with alkylating chemotherapy,^3–6^ the null hypothesis was a rate of 30% or lower (p0 = 30%). A PFS-6 ≥ 50% (p1 = 50%) was considered necessary to warrant further investigations. The sample size was based on a single-stage Fleming design with a type I unilateral error of 5% and a power of 80%. The number of subjects to be included was calculated to be 35.

### Patients

Key inclusion criteria were: Recurrent grade 3 or 4 IDHm HGGs or recurrent grade 2 IDHm glioma with histological or radiological (appearance of contrast enhancement) evidence of anaplastic transformation, recurrence after radiotherapy and at least one line of previous alkylating chemotherapy (PCV or TMZ), age ≥ 18 years, Karnofsky performance status ≥ 70, recurrence occurring ≥ 12 weeks from the end of the radiotherapy or occurring outside the irradiated volume, life expectancy ≥ 16 weeks, and radiologically measurable disease based on Response Assessment in Neuro-Oncology (RANO) criteria^12^ (except in patients who underwent a recent resurgery). There was no limitation in terms of previous recurrence, and previous treatment with bevacizumab was allowed. The IDH mutation and 1p/19q codeletion were assessed locally as well as *CDKN2A* homozygous deletion in astrocytoma patients. Histological diagnosis was retrospectively reclassified according to the WHO 2021 classification when possible, and otherwise according to the WHO 2016 classification.^13^

### Treatment

Eligible patients received oral olaparib at the dose of 300 mg twice daily, for a total daily dose of 600 mg. One cycle corresponded to 4 weeks of treatment. Treatment was continued until disease progression or unacceptable toxicity.

### Response Assessment

Radiographic tumor assessments were performed by the investigators using contrast-enhanced magnetic resonance imaging (MRI) according to the RANO criteria.^12^ The MRI protocol included at least axial T1-weighted, 3D T1-weighted post-gadolinium injection, and 3D T2-weighted or fluid-attenuated inversion recovery (FLAIR) sequences. Brain MRIs were performed at baseline and then every 8 weeks ± 7 days.

### Study Endpoints

The primary endpoint of the trial was the PFS-6 based on the RANO criteria.^12^ PFS-6 was defined as the proportion (%) of patients who remained alive without progression 24 weeks after study inclusion. PFS was defined as the interval from study inclusion to tumor progression or death due to any cause, whichever occurred first. Secondary endpoints were median PFS, median overall survival (OS), overall response rate (ORR), the longitudinal changes in health-related quality of life according to the EORTC quality of life questionnaire core-30 (QLQ-C30, version 3) and the EORTC quality of life questionnaire—brain cancer module (QLQ-BN20), and the type, frequency, and severity of adverse events (AE) and serious AE graded according to the revised NCI Common Terminology Criteria V4.03 for AE (NCI CTCAE V4.03). AE were assessed continuously during treatment and at least 30 days after the end of the treatment. Exploratory endpoints were the identification of characteristics associated with PFS-6 and PFS.

### Statistical Analyses

Quantitative variables were described with median, range, and interquartile range, and qualitative variables with frequencies and percentages. The primary endpoint was the PFS-6. This rate was given with its 95% Clopper-Pearson confidence interval. The analysis of the prognostic factors on the primary endpoint was carried out with the exact Fisher test. Data were censored at the last documented date without progression. OS was defined as the interval from inclusion to death due to any cause. Patients alive at the date of the analysis were considered censored at the last documented contact. PFS and OS were estimated using the Kaplan–Meier method with a 95% confidence interval [95%CI]. The ORR was calculated as the percentage of patients who had at least 1 partial or complete response documented during the treatment, according to RANO criteria. Duration of response was calculated as the time from confirmation of stable, partial, and complete response using RANO criteria until tumor progression. We used Cox models in order to estimate the hazard ratio (and its 95% CI) associated with prognostic factors of PFS (univariate analysis). For quality-of-life analysis, the following dimensions were preselected: Physical functioning, social functioning, global health status, motor dysfunction, and communication deficit. The evolution of quality of life over time was analyzed using a linear mixed model: A slope corresponding to a variation of 5 points in 10 weeks was considered to be clinically relevant. All tests were performed with a two-sided 0.05 level of significance. Analyses were performed using statistical software R version 4.1.0 (R Core Team (2020]).

## Results

### Patient Characteristics

From March through October 2019, 35 patients with recurrent IDHm HGGs were included, their demographic and clinical features are presented in Table 1. Patients’ individual characteristics are provided in Supplementary Table 1. All patients started olaparib after a median time of 3 days (range: 0–11) after the inclusion visit. Median age at study inclusion was 48 years (range: 25–64 years) and median Karnofsky performance status was 80% (range: 70%–100%). The median time since initial histological diagnosis was 7.5 years (range 1–22 years). In the 21 patients (60%) in whom the initial diagnosis was a grade 2 glioma, all patients demonstrated anaplastic transformation at the time of study inclusion, which was diagnosed based on radiological criteria in 11 and histology in 10 patients. 1p/19q codeletion status was available in 32/35 patients and was present in 13 patients (37%) and absent in 19 patients (63%). *CDKN2A* homozygous deletion could be assessed in the initial tumor of 17 astrocytomas (CGH arrays *n* = 12, methylation arrays *n* = 5) and was present in 4 patients. Overall, based on histological analysis at diagnosis or at recurrence in patients who underwent a new surgery (*n* = 17, median time before study inclusion: 36 months [1–204 months]), the last documented histological grade before study inclusion was grade 2 in 11 patients (31.5%), grade 3 in 11 patients (31.5%) and grade 4 in 13 patients (37%), respectively. All patients received radiotherapy and at least one line of chemotherapy and presented with contrast-enhancing tumors at study inclusion.

**Table 1.** Demographic and Clinical Features of Patients at Baseline

Individuals, *n*	35
*Sex, n (%)*	
Male	26 (74%)
Female	9 (26%)
*Age (years)*	
Median (range)	48 (25–64)
*KPS at baseline, n (%)*	
70–80	19 (54%)
90–100	16 (46%)
*Clinical presentation, n (%)*	
Focal neurological deficit	10 (29%)
Cognitive dysfunction	17 (49%)
Seizures	7 (20%)
*Time since histological diagnosis (years)*	
Median (range)	7.5 (1-21)
*Initial histological diagnosis and grade, n (%)*	
Oligodendroglioma grade 2/ grade 3	8 (24%)/ 5 (14%)
Astrocytoma grade 2*/ grade 3**/ grade 4***	13 (37%)/ 4 (11%)/ 5(14%)
*Last documented histological grade before inclusion, n (%)*	
Grade 2^%^	11 (31.5%)
Grade 3	11 (31.5%)
Grade 4	13 (37%)
*IDH mutation, *n* (%)* IDH1R132H mutation	32 (91%)
	
Noncanonical IDH mutation	3 (9%)
*1p/19q codeletion, n (%)*	
No	19 (54%)
Yes	13 (37%)
NA	3 (9%)
*Time since the end of RT (months)* Median (range)	33 (7-219)
*Number of previous CT lines, n (%)*	
1	8 (23%)
2	11 (31%)
3	10 (29%)
4 or 5	6 (17%)
*Type of previous CT lines, n (%)*	
TMZ	33 (94%)
PCV or CCNU	26 (74%)
Bevacizumab	10 (29%)
Other	9 (26%)
*Time since the end of previous treatment (months)* Median (range)	8 (0.6–102)
*Longest diameter of enhancing tumor (mm)* Median (range)	47.5 (9–125)

NA, not available; RT, radiotherapy; CT, chemotherapy; TMZ, temozolomide; * 1p/19q codeletion not available in one patient and *CDKN2A* homozygous deletion unavailable in 4 patients ** *CDKN2A* homozygous deletion unavailable in 1 patient ***1p/19q codeletion not available in 2 patients, a *CDKN2A* homozygous deletion was present in 4 and not available in 1 patient ^%^ all these patients demonstrated radiological evidence of anaplastic transformation.

### Efficacy

After a median follow-up of 10.8 months, olaparib was discontinued in 31 patients (89%) because of tumor progression. In 3 patients (9%) it was discontinued based on patient and investigator decision after 11, 17, and 28 months while they were still progression-free. In 1 patient (3%), olaparib was discontinued because of the suspicion of tumor progression after 2 cycles; however, a biopsy found no viable tumor and the patient remained progression-free for 27 months after olaparib withdrawal and no further oncological treatment. This patient was finally considered by the local investigator as having not progressed.

**Figure 1.**
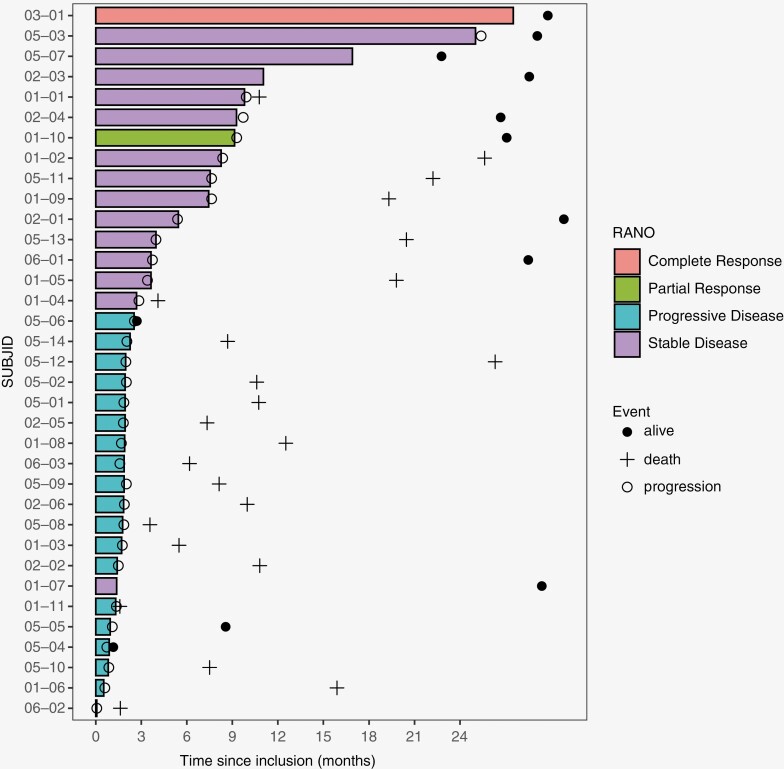
Swimmer plot showing time on therapy, time to events (progression according to RANO criteria, death), and best response (per local investigator) for each patient.

**Figure 2.**
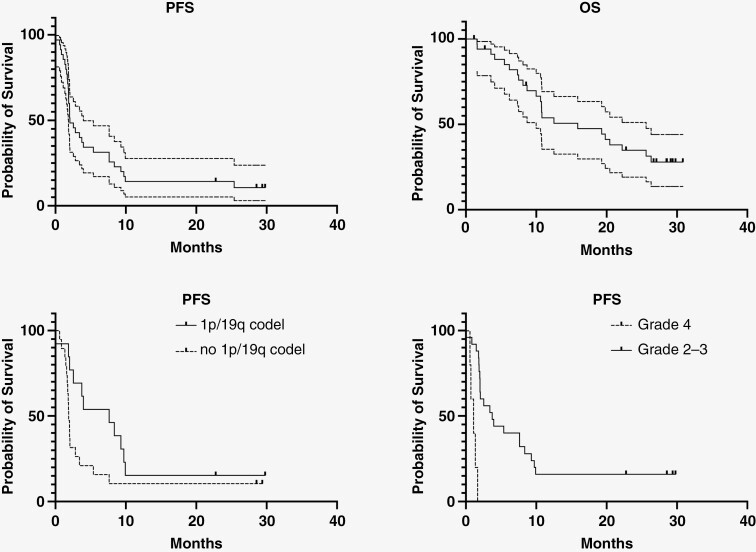
Progression-free survival and overall survival curves in the whole series (top left and right) and according to 1p/19q codeletion status and to grade at diagnosis (bottom left and right). PFS and OS in the whole series are shown with their corresponding 95% confidence interval.

At 24 weeks, 11/35 patients were progression-free resulting in a PFS-6 of 31.4% (95% CI [16.9; 49.3%]). Therefore, the study did not reach its pre-defined threshold for success (Figure 1). According to the RANO criteria, the ORR was 6%, one patient had a complete response (3%) and another had a partial response (3%, Supplementary Figure); both patients had an oligodendroglioma. The best response for 14 patients (40%) was stable disease. The median duration of response in patients with an objective response or stable disease was 6 months. Four patients (11%) were progression-free for more than 12 months after treatment initiation. The median PFS was 2.05 months (95%CI [1.85; 7.63]) and the median number of olaparib cycles was 2 (range: 1-30). The median OS was 15.9 months (CI 95%[10.6;26.3]) (Figure 2).

The presence of a 1p/19q codeletion was associated with a higher rate of PFS-6 (53.8% vs 15.8%, *P* = .05) and a trend towards a longer PFS (Hazard Ratio = 0.48, 95% CI [0.22; 1.05] *P* = .06). In contrast, patients with an initial or a last documented grade 4 astrocytoma tended to have a lower rate of PFS-6 (0% vs 36.7%, *P* = .16 and 15.4% vs 40.9%, *P* = .15, respectively) and a shorter PFS (HR = 15.6, 95% CI [3.9; 61.6], *P* < .001and HR 2.5, 95%CI [1.2; 5.1], *P* = .02) compared to patients with an initial or last documented grade 2/3 glioma (Figure 2). Neither PFS-6 nor PFS were significantly associated with median age at diagnosis, sex, presence of a neurological deficit, number of previous chemotherapy lines, time since the end of previous treatment, prior treatment with bevacizumab, and median size of contrast-enhancing lesion (Table 2).

**Table 2.** Subgroup Analysis of Primary Endpoint

Variable	Groups	PFS-6	*P* value
Age	<48 years	23.5%	.47
	≥48 years	38.9%	
Sex	Male	38.5%	.22
	Female	11.1%	
Focal neurological deficit	Yes	30%	1
	No	32%	
1p19q codeletion	Yes	53.8%	.05
	No	15.8%	
Histological grade at diagnosis	Grade 2–3	36.7%	.16
	Grade 4	0%	
Last documented histological grade*	Grade 2–3	40.9%	.15
	Grade 4	15.4%	
Number of previous CT lines	1–2	21.1%	.27
	3–5	43.8%	
Time since the end of the previous treatment	<3 months	23.1%	.48
	≥3 months	36.4%	
Previous bevacizumab	Yes	30%	1
	No	32%	
Size of the enhancing lesion at baseline**	<47.5 mm	40%	.27
	≥47.5 mm	20%	

CT, chemotherapy,*last documented histological grade: histological grade at the time of resurgery or at initial diagnosis if no resurgery was performed,** longest diameter.

### Treatment-Related AE

Five patients (14%) presented a grade 3 treatment-related AE and 15 patients (43%) a grade 2 treatment-related AE (Table 3). Grade 3 treatment-related AEs were diarrhea (*n* = 1), fatigue (*n* = 2), lymphopenia (*n* = 3), and transient aphasia (*n* = 1); the most frequent grade 2 treatment-related AEs were fatigue (26%), gastrointestinal (21%), and lymphopenia (14%). No grade 4 or 5 treatment-related AE was observed and no patient definitively discontinued olaparib due to a treatment-related AE.

**Table 3.** Reported Adverse Events Related to Olaparib

	Grade 1	Grade 2	Grade 3
*Gastrointestinal (n, %)*			
Diarrhea	1 (3%)		1 (3%)
Nausea	6 (17%)	2 (6%)	
Vomiting	2 (6%)	2 (6%)	
Abdominal pain	1 (3%)	2 (6%)	
Constipation	1 (3%)	1 (3%)	
*Constitutional (n,%)*			
Fatigue	9 (26%)	9 (26%)	2 (6%)
*Increased ALT* (*n*,%)	1 (3%)	1 (3%)	
*Hematological (n;%)*			
Lymphopenia	2 (6%)	5 (14%)	3 (9%)
Neutropenia		1 (3%)	
*Nervous system (n,%)*			
Aphasia			1 (3%)
Diziness	2 (6%)	2 (6%)	
Seizure	1 (3%)	1 (3%)	
Pain		1 (3%)	
Prurit	1 (3%)		
Headache	4 (11%)		
Number of patients (*n*,%)	20 (57%)	15 (43%)	5 (14%)

ALT, alanine aminotransferase.

### Quality of Life

Longitudinal evaluation of the quality-of-life according to QLQ-C30 and BN20 questionnaires found no significant deterioration of physical functioning (*P* = .27), social functioning (*P* = .34) and global health status (*P* = .67). There was a significant deterioration of motor dysfunction (*P* = .01) and communication deficit (*P* = .01) over time however the magnitude of the deterioration (1.6 and 2.7 points per 10 weeks, respectively) was not clinically significant.

## Discussion

In this heavily pretreated population of recurrent IDHm HGGs, olaparib monotherapy was well tolerated but did not reach the pre-defined threshold for success. Nevertheless, it resulted in some activity, particularly in patients with 1p/19q codeleted oligodendrogliomas.

The present study’s results are consistent with those of a phase 2 study that investigated the efficacy of olaparib monotherapy in 15 patients with recurrent contrast-enhancing grade 2 to 4 IDHm gliomas and which found a median PFS of 3.6 months and stable disease as the best response in 60% of the patients with a median 5.4 months disease control^10^ (as in the present study, grade 2 glioma patients who were included demonstrated evidence of anaplastic transformation). Interestingly, as in our study, patients with a grade 4 tumor (defined by histology or *CDKN2A* alteration) had a shorter PFS than those with an initial diagnosis of grade 2/3 tumors (1.8 vs. 5.23 months). Taken together, these results suggest that olaparib monotherapy may be associated with clinical benefit in a subset of patients with recurrent IDHm HGGs, particularly in those with an initial lower-grade histology. In the present study, we found that oligodendrogliomas had a more pronounced benefit than astrocytomas. In the study reported by Fanucci et al. only 3 patients had oligodendrogliomas, their median PFS was 3.6 to 4.9 months.^10^ Interestingly, inactivation of *XRCC1*, a DNA damage response gene located on 19q and that is therefore haploinsufficient in oligodendrogliomas, has been shown to render cells sensitive to PARPi.^14,15^ In future studies, it would be interesting to determine whether alterations of DNA damage response genes impact the response of IDHm gliomas to PARP inhibitors.

Nevertheless, the efficacy of olaparib herein remained modest and seems inferior to that reported for alkylating chemotherapies, although these studies were likely conducted in less heavily treated patients^3–6,16–20^ (Table 4). The efficacy of olaparib monotherapy reported herein also contrasts with what could have been expected from preclinical studies. Indeed, IDHm tumors sensitivity to PARP inhibition have been confirmed in IDHm cholangiocarcinoma and acute myeloid leukemia models.^9,21–23^ Several hypotheses could explain the discrepancy between preclinical and clinical results. Although olaparib has a poor penetration across the normal blood-brain barrier, it has been shown to achieve clinically significant concentrations in the contrast-enhancing part and the margin of recurrent glioblastomas.^24^ However, this may be suboptimal in recurrent IDHm HGGs that are generally more infiltrative and less contrast-enhanced than recurrent glioblastomas. The use of PARP inhibitors with a better brain penetration than olaparib should therefore be considered in future studies.^25^ It is also possible that too advanced IDHm gliomas, with tumors having acquired additional genetic alterations, are no longer addicted to IDH mutations and 2HG production.^26^ Indeed, targeted IDH inhibitors have been shown to be mainly effective in recurrent IDHm gliomas without contrast enhancement^7^ while in our study all patients had contrast-enhancing gliomas. Finally, although a small case series reported efficacy of olaparib monotherapy in IDHm mesenchymal sarcomas,^27^ several preclinical studies suggested that PARP inhibition in IDHm tumors was more effective when combined with temozolomide^21^ or radiotherapy.^23^ Consistent with this hypothesis, a retrospective study reported an objective response in 50% of grade 2–3 gliomas (*n* = 9) treated with olaparib and temozolomide, but no responses were observed in grade 4 IDHm astrocytomas.^28^

**Table 4.** Summary of Previous Studies in Recurrent High-Grade (IDH-Mutant) Gliomas

	Phase	*N*	Previous treatment	Median time since initial diagnosis/ since RT (months)	IDH-mt	1p/19q codel	Treatment	Response rate	Six months PFS	Median PFS (months)	Median OS (months)
Triebels, 2004^4^	2	24	RT + TMZ	—	—	68%	PCV	17%	50%	6	—
van den Bent, 2001^5^	2	27	RT + PCV	—	—	—	TMZ	26%	44%	—	7
Chinot, 2001^3^	2	48	RT + PCV	—	—	—	TMZ	44%	51%	6.7	10
van den Bent, 2003^6^	2	32	RT + PCV	−/34	—	—	TMZ	25%	29%	3.7	12.3
Soffietti, 2004^16^	2	23	RT + PCV	—	—	—	Carboplatin	13%	34.8%	3	16
van den Bent, 2018^17^	2	155	RT +/−TMZ	−/29	77%	0%	TMZ vs TMZ + bev.	32%–36%	50%–53.9%	6.1–6.9	13.8–15
Jaeckle, 2019^18^	2	51	RT + CT	−/47	NA	57%	Imatinib	3.9%	33%	4	16.6
Sepulveda-Sanchez, 2020^19^	2	34	RT + CT	115/-	100%	100%	Palbociclib	0%	21.2%	2.8	32
Mellinghoff, 2020*^7^	2	27	RT+/− CT	—	100%	33%	Ivosidenib	0%	16%	1.4	—
Picca, 2024^20^	2	39	RT + CT	68/54	100%	39%	Nivolumab	10%	28%	1.8	14.7
Schaff, 2022**^28^	R	15	RT + CT	—	100%	33%	Olaparib + TMZ	50%^%^/0%^$^	—	7.8^%^/1.3^$^	—
Fanucci, 2023^10^	2	15	RT + CT	−/66	100%	20%	Olaparib	0%	26.7%	3.6	20.7
Present study	2	35	RT + CT	90/33	100%	46%	Olaparib	6%	31%	2.05	15.9

R: retrospective case series, *only the results of patients with contrast-enhanced tumors are summarized **only results of IDH-mutant patients are summarized,^%^ and ^$^ grade 2/3 and grade 4 IDH-mutant cases, respectively.

Based on the results presented herein, olaparib monotherapy cannot be considered as a new treatment option in recurrent IDHm HGGs. Nevertheless, our study supports further research regarding the use of PARP inhibitors in IDHm gliomas. Several clinical trials are currently ongoing in IDHm gliomas to assess the safety and the efficacy of PARP inhibitors in association with temozolomide (NCT03749187), carboplatin (NCT04740190), or immune checkpoint inhibitors (NCT03991832 and NCT05188508) because PARP inhibitors exhibit immune-modulating properties in preclinical models.^29^ Indeed, PARP inhibitors could induce immunogenic cell death due to the accumulation of unrepaired double-strand breaks and have been shown to activate the stimulator of interferon genes (STING) pathway.^30,31^

Limitations of the present study include the fact that it was a non-comparative trial, the absence of central radiological review, the absence of histological confirmation of anaplastic transformation as well as the absence of 1p/19q codeletion and *CDKN2A* homozygous deletion assessment in all of the patients. Another limitation is patients’ heterogeneity. Indeed, based on the hypothesis that olaparib would be effective in IDHm HGGs irrespective of glioma subtype and initial grade, we included patients with an initial diagnosis of low or high-grade oligodendroglioma or astrocytoma. In addition, given olaparib’s expected mechanism of action in IDHm gliomas, we hypothesized that it would be effective irrespective of previous treatment which led us to include heterogeneously and heavily pretreated patients, without limit on the number of previous treatments with a median time since diagnosis of 7.5 years and 33 months since radiotherapy. It is possible that patients’ heterogeneity and heavy pretreatment precluded obtaining firm conclusions regarding olaparib’s efficacy in our study.

In conclusion, although it did not meet its primary endpoint, the OLAGLI trial demonstrated that olaparib was well tolerated and associated in some patients with prolonged responses. The present study supports further evaluation of PARP inhibitors in IDHm HGGs, especially in oligodendrogliomas.

POLA Network

Amiens (C. Desenclos, N. Guillain), Angers (P. Menei, A. Rousseau), Annecy (T. Cruel, S. Lopez), Besançon (M. Abad, N. Hamdan), Bicêtre (C. Adam, F. Parker), Brest (R. Seizeur, I. Quintin-Roué), Bordeaux (G. Chotard, C. Bronnimann), Clamart (D. Ricard), Clermont-Ferrand (C. Godfraind, T. Khallil), Clichy (D. Cazals-Hatem, T. Faillot), Colmar (C. Gaultier, MC. Tortel), Cornebarrieu (I. Carpiuc, P. Richard), Dijon (H. Aubriot-Lorton, F. Ghiringhelli), Lille (A. Djelad, CA. Maurage), Limoges (EM. Gueye, F. Labrousse), Lyon (F. Ducray, D. Meyronet), Marseille (D. Figarella-Branger, O. Chinot), Montpellier (L. Bauchet, V. Rigau), Nancy (G. Gauchotte, L. Taillandier), Nantes (M. Campone, D. Loussouarn), Nice (V. Bourg, F. Vandenbos-Burel), Nîmes (J.-S. Guillamo, P. Roger) Orléans (C. Blechet), Paris (H. Adle-Biassette, F. Bielle, A. Carpentier, C. Dehais), Poitiers (S. Milin, M. Wager), Reims (P. Colin, MD. Diebold), Rennes (D. Chiforeanu, E. Vauleon), Rouen (F. Marguet, O. Langlois), Saint-Etienne (F. Forest, MJ. Motso-Fotso), Saint-Pierre de la Réunion (M. Andraud), Strasbourg (B. Lhermitte, G. Noel), Suresnes (M. Bernier, N. Younan), Tours (C. Rousselot-Denis, I. Zemmoura), Toulon (C. Joubert), Toulouse (E. Cohen-Moyal, E. Uro-Coste), and Villejuif (F. Dhermain).

## Supplementary Material

vdae078_suppl_Supplementary_Materials
