# Supplementary material for: Olaparib in recurrent isocitrate dehydrogenase mutant high-grade glioma: A phase 2 multicenter study of the POLA Network
Source: Neurooncol Adv. 2024 May 27;6(1):vdae078. doi: 10.1093/noajnl/vdae078 (PMC11157627; doi:10.1093/noajnl/vdae078)
Supplement: vdae078_suppl_Supplementary_Materials [file vdae078_suppl_supplementary_materials.docx]

**Supplementary Table : individual patients’ characteristics**

| **SUBJID** | **IDH mutation** | **1p/19q codel  (initial)*** | **1p/19q codel  (final)**** | **CDKN2A homo.  deletion** | **WHO 2016  diagnosis** | **WHO 2021  diagnosis** | **Initial grade** | **Resurgery** | **Last documented  histological grade** | **Previous CT  lines** | **Type of previous CT** | **Best  response** | **PFS6** |
| --- | --- | --- | --- | --- | --- | --- | --- | --- | --- | --- | --- | --- | --- |
| 01-01 | IDH1 R132H | Yes | Yes | NA | O2 | O2 | 2 | No | 2 | 4 | TMZ, PCV, TMZ, BEV+IRI | SD | Yes |
| 01-02 | IDH1 R132H | Yes | Yes | NA | O2 | O2 | 2 | Yes | 3 | 3 | PCV, Carmustine w, TMZ | SD | Yes |
| 01-03 | IDH1 non canonical mutation | No | No | NA | A2 | NA | 2 | Yes | 4 | 3 | PCV, TMZ, Carboplatine | PD | No |
| 01-04 | IDH1 R132H | No | No | NA | A2 | NA | 2 | Yes | 2 | 5 | CCNU+TMZ, TMZ, TMZ, BEV+CCNU,  BEV | SD | No |
| 01-05 | IDH1 R132H | No | No | No | A3 | A3 | 3 | No | 3 | 4 | PCV, TMZ, | SD | No |
| 01-06 | IDH1 R132H | No | No | Yes | A4 | A4 | 4 | No | 4 | 2 | TMZ, BEV | PD | No |
| 01-07 | IDH1 R132H | No | No | No | A2 | A2 | 2 | No | 2 | 4 | TMZ, TMZ,PCV, BEV | SD | Yes |
| 01-08 | IDH1 R132H | No | No | Yes | A3 | A4 | 4 | No | 4 | 2 | BEV, TMZ | PD | No |
| 01-09 | IDH1 R132H | No | Yes | Yes | O3 | O3 | 3 | Yes | 3 | 3 | TMZ, Carmustine w, PCV | SD | Yes |
| 01-10 | IDH1 R132H | NA | Yes | No | O3 | O3 | 3 | Yes | 3 | 3 | PCV, TMZ, Carmustine w | PR | Yes |
| 01-11 | IDH1 R132H | No | No | Yes | A4 | A4 | 4 | No | 4 | 2 | TMZ, CCNU+BEV | PD | No |
| 02-01 | IDH1 R132H | NA | No | No | A3 | A3 | 3 | No | 3 | 3 | TMZ, CCNU, BEV | SD | No |
| 02-02 | IDH1 R132H | Yes | No | No | A2 | A2 | 2 | Yes | 4 | 1 | TMZ | PD | No |
| 02-03 | IDH1 R132H | No | No | No | A3 | A3 | 3 | Yes | 4 | 2 | TMZ, BEV+IRI | SD | Yes |
| 02-04 | IDH1 R132H | Yes | Yes | No | O3 | O3 | 3 | Yes | 3 | 3 | TMZ, TMZ, PCV | SD | Yes |
| 02-05 | IDH1 R132H | No | No | No | A2 | A2 | 2 | Yes | 3 | 4 | PCV, TMZ+BEV, TMZ,  Fotemustine | PD | No |
| 02-06 | IDH1 R132H | Yes | No | No | A2 | A2 | 2 | Yes | 3 | 2 | TMZ, CCNU | PD | No |
| 03-01 | IDH1 R132H | Yes | Yes | NA | O3 | O3 | 3 | No | 3 | 3 | TMZ, TMZ, PCV | CR | Yes |
| 05-01 | IDH1 R132H | Yes | Yes | Yes | O2 | O2 | 2 | No | 2 | 1 | PCV | PD | No |
| 05-02 | IDH1 R132H | Yes | Yes | No | O2 | O2 | 2 | No | 2 | 2 | TMZ, PCV | PD | No |
| 05-03 | IDH1 R132H | NA | NA | NA | A2 | NA | 2 | No | 2 | 1 | TMZ | SD | Yes |
| 05-04 | IDH1 R132H | Yes | NA | Yes | A4 | A4 | 4 | No | 4 | 1 | TMZ, TMZ | PD | No |
| 05-05 | IDH1 R132H | NA | NA | NA | A4 | A4 | 4 | No | 4 | 1 | TMZ | PD | No |
| 05-06 | IDH2 mutation | Yes | Yes | NA | O2 | O2 | 2 | No | 2 | 1 | PCV | PD | No |
| 05-07 | IDH2 mutation | Yes | Yes | No | O3 | O3 | 3 | No | 3 | 2 | TMZ, PCV | SD | Yes |
| 05-08 | IDH1 R132H | Yes | No | No | A2 | NA | 2 | Yes | 4 | 2 | PCV, TMZ | PD | No |
| 05-09 | IDH1 R132H | Yes | No | No | A2 | A2 | 2 | Yes | 4 | 3 | TMZ, PCV, Carboplatine | PD | No |
| 05-10 | IDH1 R132H | No | No | No | A2 | A2 | 2 | Yes | 3 | 2 | TMZ, CCNU | PD | No |
| 05-11 | IDH1 R132H | No | No | No | A2 | A2 | 2 | Yes | 4 | 2 | TMZ, PCV | SD | Yes |
| 05-12 | IDH1 R132H | NA | No | No | A2 | A2 | 2 | Yes | 4 | 1 | TMZ | PD | No |
| 05-13 | IDH1 R132H | Yes | Yes | No | O2 | O2 | 2 | No | 2 | 1 | TMZ | SD | No |
| 05-14 | IDH1 R132H | No | No | No | A2 | A2 | 2 | Yes | 2 | 3 | TMZ, TMZ, CCNU | PD | No |
| 06-01 | IDH1 R132H | Yes | Yes | NA | O2 | O2 | 2 | No | 2 | 2 | TMZ, PCV | SD | No |
| 06-02 | IDH1 R132H | Yes | Yes | NA | O2 | O2 | 2 | No | 2 | 5 | TMZ, TMZ, PCV, Carboplatine | PD | No |
| 06-03 | IDH1 R132H | No | No | NA | A3 | NA | 3 | Yes | 4 | 3 | TMZ, PCV, BEV | PD | No |

O2/3 : oligodendroglioma grade 2/3; A2/3/4 : astrocytoma grade 2/3/4; *locally assessed 1p/19q codeletion status was available at inclusion in 30 patients and considered as present in 16 patients and absent in 14 patients; **after complementary molecular analyses 1p/19q codeletion status could be determined in 2 additional patients and was revised 6 patients; types of previous chemotherapy: TMZ: temozolomode, PCV (Procarbazine, CCNU, Vincristine), BEV: bevacizumab, IRI: irinotecan, Carmustine w: carmustine wafers; PFS6, yes: patient progression free at 24 weeks, no: patient who progressed before week 24.

**Supplementary Figure**

Patient 01-010: diagnosis of grade 3 oligodendroglioma in 1997 treated with PCV (1997), RT (2008), TMZ (2012) and resurgery plus Carmustine wafers in 2013 (re-surgery: oligodendroglioma grade 3 IDH1R132H mutant 1p/19q codeleted). Flair progression and new contrast-enhanced lesion leading to inclusion in the OLAGLI trial in July 2019 until progression in May 2020 and resurgery demonstrating a recurrence of the grade 3 oligodendroglioma. Baseline: MRI before olaparib start.
